# Definition of Metrics to Evaluate Cochlear Array Insertion Forces Performed with Forceps, Insertion Tool, or Motorized Tool in Temporal Bone Specimens

**DOI:** 10.1155/2014/532570

**Published:** 2014-07-15

**Authors:** Yann Nguyen, Guillaume Kazmitcheff, Daniele De Seta, Mathieu Miroir, Evelyne Ferrary, Olivier Sterkers

**Affiliations:** ^1^Otolaryngology Department, Unit of Otology, Auditory Implants and Skull Base Surgery, Hospital Pitié Salpêtrière, 47-83 Boulevard de l'Hôpital, Cedex 13, 75651 Paris, France; ^2^Sorbonne University, “Minimally Invasive Robot-Based Hearing Rehabilitation”, UPMC Univ Paris 06, UMR S 1159, 75005 Paris, France; ^3^INSERM, “Minimally Invasive Robot-Based Hearing Rehabilitation”, UMR S 1159, 75018 Paris, France; ^4^Sensory Organs Department, Sapienza University of Rome, 00100 Rome, Italy

## Abstract

*Introduction*. In order to achieve a minimal trauma to the inner ear structures during array insertion, it would be suitable to control insertion forces. The aim of this work was to compare the insertion forces of an array insertion into anatomical specimens with three different insertion techniques: with forceps, with a commercial tool, and with a motorized tool. *Materials and Methods*. Temporal bones have been mounted on a 6-axis force sensor to record insertion forces. Each temporal bone has been inserted, with a lateral wall electrode array, in random order, with each of the 3 techniques. *Results*. Forceps manual and commercial tool insertions generated multiple jerks during whole length insertion related to fits and starts. On the contrary, insertion force with the motorized tool only rose at the end of the insertion. Overall force momentum was 1.16 ± 0.505 N (mean ± SD, *n* = 10), 1.337 ± 0.408 N (*n* = 8), and 1.573 ± 0.764 N (*n* = 8) for manual insertion with forceps and commercial and motorized tools, respectively. *Conclusion*. Considering force momentum, no difference between the three techniques was observed. Nevertheless, a more predictable force profile could be observed with the motorized tool with a smoother rise of insertion forces.

## 1. Introduction

Cochlear implant is a neural prosthesis that is inserted within the cochlea into the scala tympani in order to electrically stimulate spiral ganglion fibers from the auditory nerve. It has become the most efficient device to rehabilitate patients suffering from severe to profound deafness [1]. Three critical steps can be identified in the cochlear implantation procedure: approach to cochlea, cochlea opening, and array insertion. Minimizing trauma during the cochlear implantation procedure is critical to preserve residual hearing in case of acoustic electric stimulation or remaining inner ear structures in case of electric stimulation only [2]. Even though multiple approaches can be performed to access cochlea such as suprameatal, transcanal, or minimally invasive key-hole access, the routine exposure of the cochlea in a vast majority of cochlear implant centers is mastoidectomy followed by posterior tympanotomy [3]. The cochlea opening through the round window membrane, a cochleostomy, or an extended round window approach remains a current debate frequently discussed [4]. These two first steps determine the axis and the entry point of the array into the cochlea. Considering solutions to reduce trauma during the array insertion, most studies compared array designs [5] and evaluated histological trauma [6] or insertion forces [7]. Even though the insertion technique remains critical for inner ear structure preservation to achieve insertion with minimal trauma, it is seldom studied. The insertion technique will be influenced by tremor, insertion speed, and duration and possibilities of insertion axis modification including torque around the array body. It is usually performed manually using forceps, microforceps, or a dedicated tool depending on the array design. Arrays including a stylet can offer various insertion techniques depending on stylet removal timing [8]. Speed of insertion or use of lubricant have also been studied and have been shown to influence frictions forces [9, 10]. Manual insertion with forceps has been compared to robotic insertion [11] but has never been compared to the insertion with other technique with specific tool. Motorization of the tool could also be employed to reduce fits and start inherent to manual insertion as it is hard to insert the array in a single move with forceps grasping. The goal of the present work was to compare cochlear array insertion forces performed by forceps, an insertion tool, or a motorized tool in temporal bone specimens with the same array design.

## 2. Material and Methods

### 2.1. Human Temporal Bone Preparation

**Figure 1:**
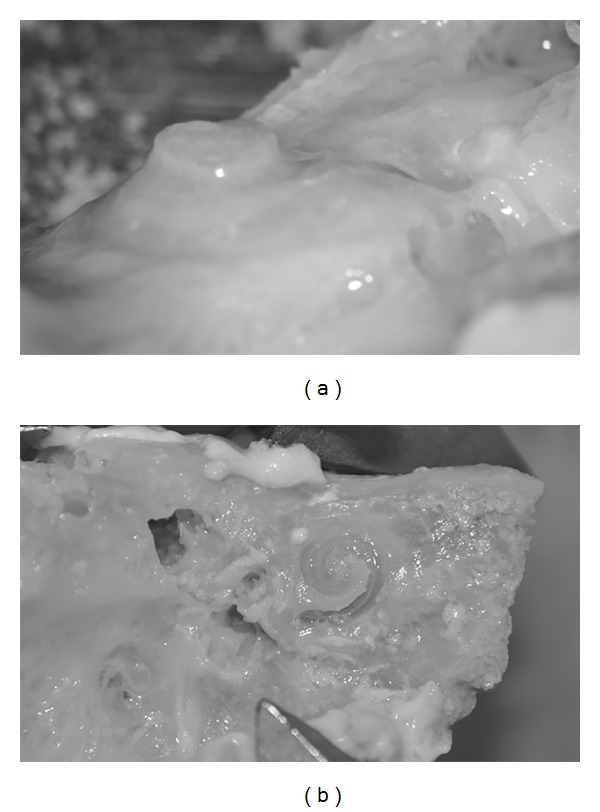
Microdissected cochlea model. (a) A wide canal wall down mastoidectomy is performed to expose the cochlea. The otic capsule is then thinned with a diamond burr (left cochlea). (b) The scalae vestibuli and media are then carefully opened to expose the basilar membrane leaving the scala tympani intact (right cochlea).

**Figure 2:**
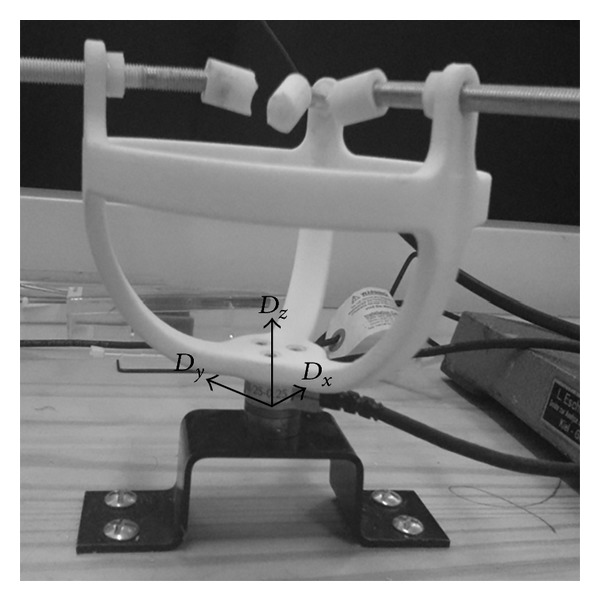
Insertion force measurement setup. A plastic temporal bone holder was screwed on a 6-axis force sensor (ATI Nano 17, Apex, NC) to record array insertion forces into a temporal bone.

Twenty human temporal bones have been prepared. The cochlea has been exposed through a canal wall down mastoidectomy. A large approach has been chosen to ease bony otic capsule drilling and avoid direct contact of the forceps, insertion tool, or motorized tool with the temporal bone. The bony otic capsule has been thinned using diamond burrs under microscope (Figure 1(a)). The scala vestibuli and the scala media have then been carefully opened taking care to respect the basilar membrane integrity from the round window to the apex (Figure 1(b)). This allowed visualization of the array progression during its insertion by transparency through the basilar membrane. This also allowed checking basilar membrane integrity and the lack of scalae translocation during insertion. An extended round window cochleostomy has then been drilled in the inferior rim of the round window. Temporal bones were then mounted on an in-house made temporal bone holder that could be fixed to a force sensor (Figure 2). The temporal bones specimens, fixed on the force sensor, have been oriented to align the array insertion axis, the scala tympani midline, and the D_*z*_ axis of the 6-axis force sensor. Cochleostomy was irrigated with saline serum before each insertion.

### 2.2. Electrode Array

Hifocus 1J arrays (Advanced Bionics, Valencia, CA) have been used in this study. 1J array is a lateral wall positioning array bearing 16 electrodes. A silicon jog is placed at its base in order to push the array with an insertion tool. This jog slides into the insertion tube and serves as the contact point for array propulsion inside the insertion tube by a rod. The array has a total length of 25 mm from the jog to the tip, an active length of 17 mm, a proximal diameter of 0.8 mm, and a distal diameter of 0.4 mm. Each array was used for two insertions and then discarded.

### 2.3. Insertion Protocol and Insertion Force Measurements

Frictions forces between the array and the cochlea have been recorded with a 6-axis force sensor (ATI Nano 17, calibration type SI-12-0.12, resolution: 3 mN, Apex, NC). Sensor data have been recorded in real-time via the same analog to digital interface card controlling the actuator input power at a sample rate of 100 Hz. From the 6-axis sensor, insertion forces were computed only based on linear force norms (D_*x*_, D_*y*_, D_*z*_).

**Figure 3:**
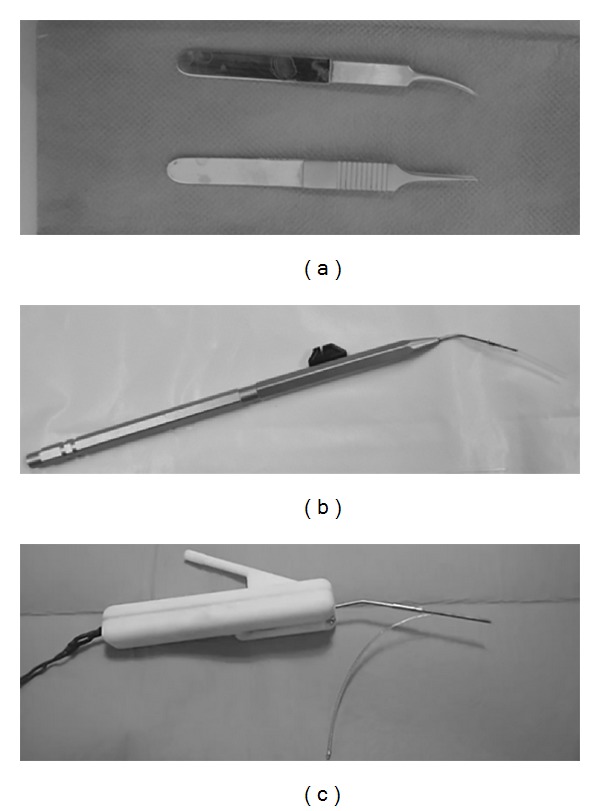
Tools and devices used in this study to insert array on temporal bones. (a) Microforceps claws, (b) Hifocus 1J tool (Advanced Bionincs, Valencia, USA), and (c) insertion with an in-house motorized tool.

Three insertion tools and techniques were randomly tested:

- manual insertion using two microforceps claws (Figure 3(a));
- insertion with the Hifocus 1J electrode insertion tool (Figure 3(b)), a commercially available tool distributed with Hifocus 1J and helix array; it is composed of a handle comprising a flexible shaft connected to a slide that can eject out of an insertion tube the array by pushing its silicon jog. We have been using the metal insertion tube (AB-6135, Advanced bionics, Valencia, CA) in this study. This tool was held manually during insertion;
- insertion with an in-house made motorized insertion tool (Figure 3(c)). This tool comprised a rotary actuator (RE10CLL, MDP, Miribel, France) connected to a threaded screw that pushed a blunt pin into an insertion tube loading the array. The tool was held steady by a flexible arm. No force feedback loop between this tool and the force sensor was applied. The actuator speed was controlled via laboratory power supply and set at 0.8 mm*·*s^−1^.

During each insertion, a particular attention was made to avoid touching directly the temporal bone with forceps or insertions tools in order to avoid artefact recording of the force sensor. For the manual and commercial tool techniques, the operator's hands were supported on a flexible arm with a metal bar similarly to a Yasargil bar as it has been shown that supporting the wrists significantly decreases the amplitude of the tremor [12]. Force measurement was coupled to video recording through the microscope to collect force data from the beginning to the end of the insertion only.

Each temporal bone was inserted three times with the three different insertions techniques in an order that was randomized. If a basilar membrane perforation occurred during insertion, the temporal bone was excluded for analysis.

### 2.4. Metrics Analysis

We investigated the shape of the curve corresponding to the force versus the time. In order to do so, we have built different metrics.

(i) The peak of force applied during the insertion: this metric quantifies a potential damage of the cochlea if an excessive force is applied. Thus, the study of the peak of force allows us to identify if an insertion method may guaranty a lower maximal force.
(ii) The total change in momentum (*I*, in  Ns) was produced during the insertion, measured by *I* = ∫*F*(*t*)*dt*.
(iii) The number of occurrence (Th) where the applied forces were over an arbitrary threshold, fixed at 0.1 N that may yield to severe damage of anatomical structure within the cochlea: this threshold value corresponds to the peak force at the end of a complete insertion of array in temporal bones from previously published data [7].
(iv) The number of times (*G*) where forces (*F*) were increased by 50% (sudden rise) within a small time step *h* = *t* − (*t* − 1) = 0.1 s: it corresponded to the number of local discontinuities of the applied forces and possibly to the number of potential local damages into the cochlea. Consider
  (1) $$\begin{array}{c} G_{t}=\{\begin{array}{cc} G_{t}-1+1, & \text{if}\;\;\frac{F_{t}}{F_{t}-1}\geq 2,\\ G_{t}-1, & \text{otherwise}. \end{array} \end{array}$$
(v) The smoothness of the curve, studied as “jerk” variation (*J*) (expressed as N*·*s^−1^): it is obtained from the derivative of the force over the time. A root mean square (RMS)  function was used to analyze the jerk variation. Consider
  (2) $$\begin{array}{c} \text{RMS}=\sqrt{\;\frac{1}{n}\overset{n-1}{\underset{i=0}{\sum }}J_{i}^{2}},\;\text{with}\;\;J=\frac{dF}{dt}. \end{array}$$

### 2.5. Statistical Analysis

Results are expressed as mean ± standard deviation. Data were analyzed and graphics were generated by “R” statistical software (http://www.r-project.org/). Comparisons between different insertions conditions were tested by ANOVA and results are presented with the associated *P* value for significant data.

## 3. Results

### 3.1. Data Collection

A basilar membrane perforation occurred in 7 temporal bones (35%) out of 20. This occurred once with a forceps insertion, 3 times with the Hifocus 1J electrode insertion tool, and 3 times with the motorized insertion. The translocation rate has to be analyzed with precaution due to model preparation. While giving immediate information during insertion on array translocation, this kind of microdissected model has the drawback of potentially creating histological damages or weakening of the basilar membrane before array insertion [7].

Thus, the implants were inserted three times in the same cochlea in 13 temporal bones (39 insertions). We investigate the possible lesions of the cochlea undergone during the first insertions, in order to determine the presence of a systematic diminution of forces for the second or the third insertions. We found that force peaks of the motorized insertion on third position were significantly different compared to measurement of first and second insertion (*P* = 0.0362). Thus, third insertion could not be used for analysis and all data collected during the third insertion were discarded in all temporal bones. Consequently, insertions forces data were used for analysis in 10 manual insertions, 8 Hifocus 1J electrode insertion tool insertions, and 8 motorized insertions.

### 3.2. Insertion Force Profiles

**Figure 4:**
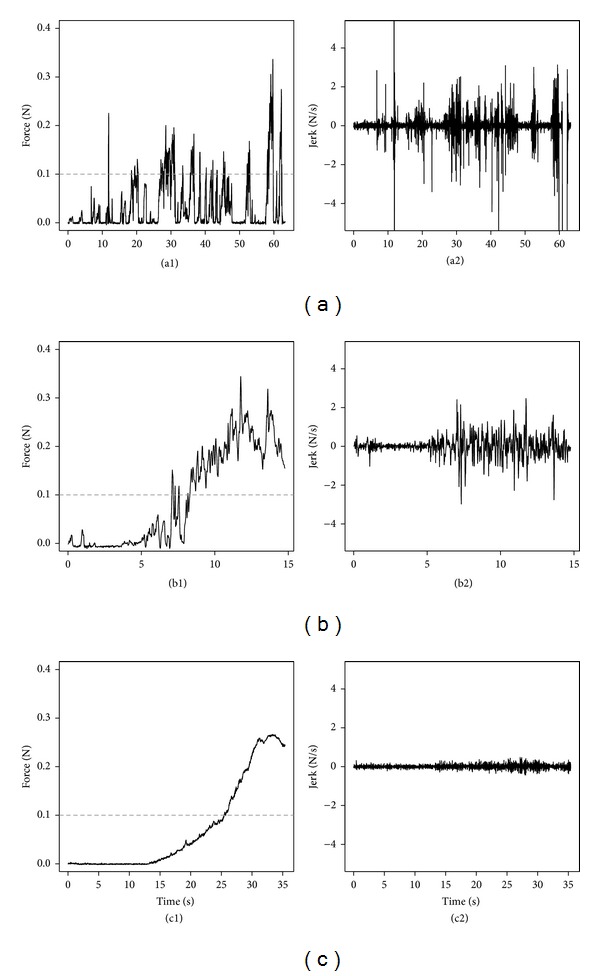
Insertion force profile and jerk of a 1J array with 3 different insertion techniques in the same temporal bone. (a) Manual insertion with microforceps claws tool, (b) insertion with Hifocus 1J electrode insertion tool, and (c) insertion with an in-house motorized tool. Left pictures ((a1), (b1), and (c1)): insertion forces profiles. Dashed line represents 0.1 N threshold. Peak forces were around 0.3 N for the three insertion techniques. Right pictures ((a2), (b2), and (c2)): jerk. Hifocus 1J electrode insertion tool provided smoother insertion with little jerk compared to manual insertion with forceps. This benefit is even more increased with a motorized tool.

Insertion force profiles had a similar shape from one temporal bone to another depending on the insertion technique. With manual forceps insertion technique (Figure 4(a1)), insertion forces remained low in the first half of the insertions with some peaks corresponding to fits and starts when the array was grasped and released multiple times from distal to proximal parts. The amplitude of these peaks rose towards the end of the insertion.

**Figure 5:**
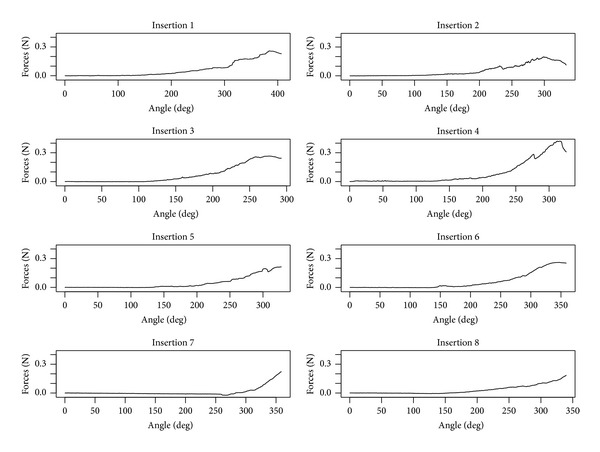
Plot using force versus angle representing insertions with the motorized tool. Insertion forces remain low in the first half on the insertion and then slowly rise with no peak and reach a maximum at the end of the insertion.

With motorized tool technique, insertion forces remained also low in the first half of the insertion (Figure 4(c1)). It rises slowly afterwards continuously without peak and reached a maximum at the end of the insertion. A plot using force versus angle representing insertions with the motorized tool is represented on Figure 5.

With the Hifocus 1J electrode insertion tool technique (Figure 4(b1)), a mix between the two previously described force profiles was observed with small amplitude peaks distributed along a force profile curve that slowly rises from the second part of the insertion toward the end.

### 3.3. Metric Analysis

**Figure 6:**
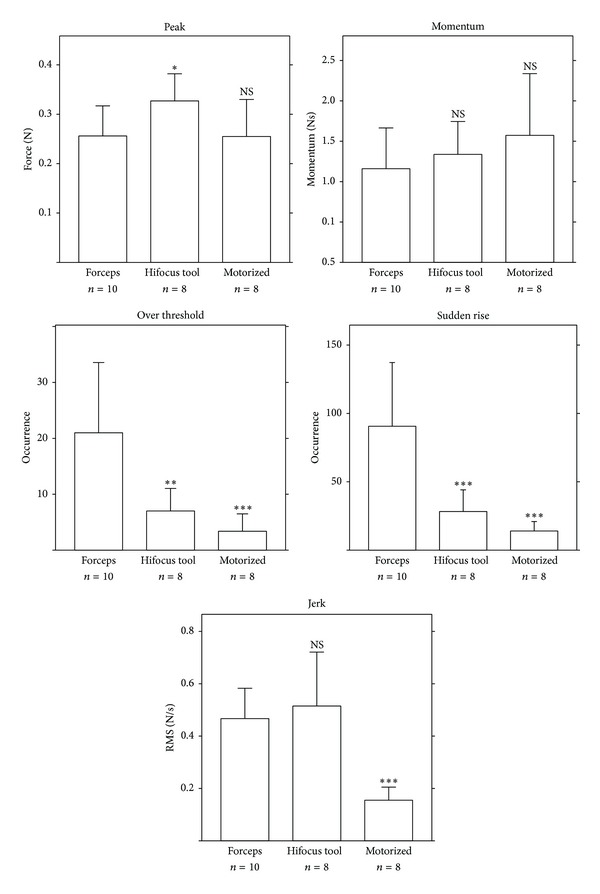
Comparison of insertion forces of cochlear implantation with 5 metrics. Bars represent mean ± SD of n insertions. NS: not significant. “Forceps” stands for manual insertion with forceps technique, “Hifocus tool” stands for Hifocus 1J electrode insertion tool technique, and “motorized” stands for our in-house motorized insertion tool technique. Statistical analysis was performed by analysis of variance. Each technique was compared against the manual insertion with forceps technique.

The results from metric analysis are reported in Figure 6 and Table 1. Considering the peak force at the end of the insertion, the Hifocus 1J electrode insertion tool had higher values than techniques with forceps and motorized tool. The momentum was the same for the three techniques. There was less threshold crossing over 0.1 N with the motorized tool compared to Hifocus 1J electrode insertion tool and the forceps manual technique. Sudden rises and jerks happened also less frequently with the motorized tool compared to manual insertion and Hifocus 1J electrode insertion tool (Figures 4(a2), 4(b2), and 4(c2)).

## 4. Discussion

In this study, we compared cochlear array insertion forces performed manually with forceps, an insertion tool, or a motorized tool in temporal bone specimens with the same array design. We have shown that there was no difference between the three techniques for peak force and total force value. A more predictable insertion force curve with less peak and rises was seen with the motorized tool compared to the two other insertion tools.

### 4.1. Advantages and Drawbacks of the Three Insertion Techniques

Each of the three techniques has advantages and drawbacks. Manual insertion with forceps is commonly used because it is compatible with most of the clinically available array device, especially straight arrays. One claw forceps is used to push the array while the other is used to guide the insertion axis. Depending on array length and stiffness, full insertion of the array cannot always be performed in single step and may require multiples grasps to insert the whole array, segment after segment. These fits and starts during the insertion procedure might generate multiple short peak forces during insertion as we observed in the present study. Resistance feedback can be perceived once a physiological threshold is reached. The force feedback sensitiveness depends on wearing gloves and is clearly subject to variability between surgeons. Furthermore this technique is subject to human limitation in terms of accuracy and tremor [13].

Insertion with the Hifocus 1J electrode insertion tool is only possible with 1J and Helix arrays because it requires a silicon jog on the array. It offers an increased stability as the insertion tube can be leant on the posterior part of the posterior tympanotomy during array insertion. The tool only requires one hand to function, thus the second hand can be used as a stabilizer to further reduce tremor. Drawbacks are represented by a lack of resistance feedback feeling because friction forces within the tool and insertion tube might interfere with surgeon sensitiveness on friction forces within the cochlea [14]. Furthermore, due to insertion tube diameter, vision of the cochleostomy or round window can be reduced a little compared to a manual forceps technique. At last the stroke of the slide of the tool can require a two-step push depending on the finger (thumb or forefinger) that is used to push the slide thus generating a fit and start during insertion.

Insertion with our current version of in-house motorized tool is only possible with 1J and helix because it requires a jog on the device to push the array. It provides a smooth and low speed insertion. Complete insertion can be achieved in a single step. Human hand tremor is removed as the tool is held by a flexible arm. However, force feedback is completely impaired and surgeon can only rely on visual control at the cochleostomy to detect a blockage within the cochlea that will lead to array bending at its proximal part outside the cochlea. Vision is also impaired just as with the tool from the second technique.

### 4.2. Previous Work on Insertion Technique Comparison and Definition of New Metrics to Study Insertion Forces

Majdani et al. had compared robotic to manual insertion with array using an advance off stylet technique in an artificial model of scala tympani [11]. It has been shown that a greater variability of frictions forces could be observed with a manual array insertion technique with more peaks compared to a robot-based insertion technique. The average force was also compared showing increased force for the robotic insertion compared to manual insertions.

We decided to define new metrics to study and compare insertion forces profiles because average force seems hard to interpret. For example manual insertion with a long duration will necessarily have a lower average force since during pauses for the duration of insertion, there is no effort on the cochlea. We could have compared the technique using forces in Newton versus angle or length of insertion. These data are easy to collect with a constant speed insertion such as the motorized tool but hard to collect with manual and Hifocus 1J electrode insertion tool because the array progression cannot be visualized through the basilar membrane as well as a transparent artificial model of scale tympani and the insertion speed during array progression with manual technique is difficult to collect. One of the limitations of this study is that we were not able to control or measure the insertion speed when using the manual or commercial tool technique.

No force sensor was mounted on the motorized insertion tool. Thus insertions with this tool were not force feedback controlled. Frictions of the array within the insertion tube of the commercial tool could impair surgeon's force feedback feeling. Thus, force feedback could only be perceived with the manual technique. This might account for the different basilar membrane perforation rates among the three techniques.

The new metrics that we have defined can help forces profiles analyses by giving absolute values such as the peak force or the forces momentum but also information on sudden forces changes or rises.

## 5. Conclusion and Perspectives

We have validated the use of metrics such as peak force, momentum of the force, threshold crossing over 0.1 N, sudden rises, and jerks that could be indicators of the quality of surgical gesture during cochlear implantation. The analysis of these metrics in insertion allows demonstrating that the Hifocus 1J electrode insertion with a commercial guided tool has less threshold crossing over 0.1 N and sudden rises compared to a manual insertion performed with forceps. These drawbacks are even more reduced with a motorized tool leading to a smoother insertion. Next step will be to introduce a force feedback control loop between the motorized tool and the force sensor in order to reduce the insertion peaks (in amplitude and in duration) and to stop the insertion in case of abnormal force sudden rise. If those parameters can be controlled, it should be possible to enhance the safety of cochlear implantation.

## Figures and Tables

**Table 1:** Metric values recorded during of the cochlear implantation with three different insertion techniques.

Metric	Insertion technique	Mean ± SD	*n*	*P*
Peak (N)	Forceps	0.256 ± 0.061	10	NA
	Hifocus tool	0.327 ± 0.055	8	0.028
	Motorized	0.255 ± 0.075	8	NS

Momentum (Ns)	Forceps	1.16 ± 0.505	10	NA
	Hifocus tool	1.337 ± 0.408	8	NS
	Motorized	1.573 ± 0.764	8	NS

Over threshold >0.1 N	Forceps	21.00 ± 12.552	10	NA
	Hifocus tool	7.00 ± 4.036	8	0.002
	Motorized	3.38 ± 3.113	8	0.0002

Sudden rise	Forceps	90.60 ± 46.569	10	NA
	Hifocus tool	28.25 ± 15.872	8	0.0003
	Motorized	14.00 ± 6.949	8	0.00003

Jerk (N*·*s^−1^)	Forceps	0.467 ± 0.116	10	NA
	Hifocus tool	0.515 ± 0.206	8	NS
	Motorized	0.1553 ± 0.05	8	0.00008

Values are expressed as mean ± SD of *n* insertion. NA: not applicable; NS: not significant. “Forceps” stands for manual insertion with forceps technique, “Hifocus tool” stands for Hifocus 1J electrode insertion tool technique, and “motorized” stands for our in-house motorized insertion tool technique. Statistical analysis was performed by analysis of variance. Each technique was compared against the manual insertion with forceps technique.
